# Association between *Helicobacter pylori cagA*-related genes and clinical outcomes in Colombia and Japan

**DOI:** 10.1186/1471-230X-11-141

**Published:** 2011-12-22

**Authors:** Masahide Watada, Seiji Shiota, Osamu Matsunari, Rumiko Suzuki, Kazunari Murakami, Toshio Fujioka, Yoshio Yamaoka

**Affiliations:** 1 Department of Environmental and Preventive Medicine, Oita University Faculty of Medicine, 1-1 Idaigaoka, Hasama-machi, Yufu-City, Oita 879-5593, Japan; 2 Department of General Medicine, Oita University Faculty of Medicine, 1-1 Idaigaoka, Hasama-machi, Yufu-City, Oita 879-5593, Japan; 3 Department of Medicine-Gastroenterology, Baylor College of Medicine and Michael E. DeBakey Veterans Affairs Medical Center, 2002 Holcombe Blvd., Houston, Texas 77030 USA

## Abstract

**Background:**

Specific genotypes of several virulence factors of *Helicobacter pylori *(eg, *cagA*-positive, *vacA *s1, *oipA *"on" and *babA*-positive) have been reported to be predictors of severe clinical outcomes. Importantly, the presence of these genotypes correlates with each other. We hypothesized that novel virulence genes correlate with the presence of *cagA*. Therefore, we aimed to find novel candidate virulence genes that correlate with *cagA *and examined the association of these genes with clinical outcomes in Colombian and Japanese populations.

**Methods:**

*cagA*-associated genes were selected based on previous *H. pylori *genome microarray data. A total of 343 strains (174 from Colombia and 169 from Japan) were examined for the status of *cagA, vacA*, and candidate genes by polymerase chain reaction and dot blot.

**Results:**

Microarray data showed that 9 genes were significantly correlated with the presence of *cagA*. Among the 9 genes, the functions of 4 were known, and we selected these 4 genes as candidate genes (*hp0967, jhp0045, jhp0046*, and *jhp0951*). The prevalences of *cagA, vacA *s1/m1 genotype, and *hp0967 *were significantly higher in Japan than Colombia, whereas those of *jhp0045 *and *jhp0046 *were more prevalent in Colombia than Japan. The prevalences of *jhp0045 *and *jhp0046 *in *cagA*-positive cases of gastric cancer were significantly higher than those from gastritis in Colombia (P = 0.015 and 0.047, respectively). In contrast, the prevalence of 4 candidate genes was independent of clinical outcomes in Japan.

**Conclusions:**

*jhp0045 *and *jhp0046 *might be novel markers for predicting gastric cancer in *cagA*-positive cases in Colombia, but not in Japan.

## Background

*Helicobacter pylori *(*H. pylori*) infection is now accepted as the major cause of chronic gastritis. In addition, several epidemiological studies have shown that *H. pylori *infection is linked to severe gastritis-associated diseases, including peptic ulcer and gastric cancer (GC) [1]. In 1994, the International Agency for Research on Cancer categorized *H. pylori *infection as a group I carcinogen [2]. Although GC is one of the most common cancers, only a minority of individuals with *H. pylori *infection ever develop it. The prevalence of GC is approximately 3% in *H. pylori*-positive patients [3].

In addition to environmental factors (eg, diet) and host factors, virulence factors of *H. pylori*, such as *cagA, vacA, oipA, babA, hopQ*, and *homA/B*, have been demonstrated to be predictors of gastric atrophy, intestinal metaplasia, and severe clinical outcomes [4-10]. The most studied virulence factor of *H. pylori *is *cagA*, which is located at the end of an approximately 40-kb cluster of genes called *cag *pathogenicity island (PAI). *cag *PAI encodes a type-IV secretion system and transfers CagA protein into host cells [11]. CagA protein is believed to have oncogenic potential [12,13], and *cagA*-positive strains are reported to be associated with severe clinical outcomes [14].

However, these factors are not enough to distinguish markers for severe outcomes (eg, GC) in Japan because most *H. pylori *strains isolated in Japan possess these virulence factors. Likewise, our previous report showed that these genes were not virulence markers for GC in Colombia [10]. Importantly, the presence of these genotypes correlate with each other; the *cagA*-positive strain usually possesses the *vacA *s1/m1 genotype, and it is further closely linked to the presence of *babA *and *oipA *"on" status [14]. Therefore, we hypothesized that novel virulence genes correlate with the presence of *cagA*. Although *cagA *is not a distinguishing marker for severe outcomes in Japan and Colombia, the importance of *cagA *has been shown in both in vitro and in vivo experiments [14]. For example, our study showed that histological scores were significantly higher in *cagA*-positive subjects than *cagA*-negative ones, even in Japan [15]. Therefore, subjects infected with *cagA*-positive *H. pylori *can be considered as a higher risk population than those with *cagA*-negative strains, even in Japan and Colombia. However, only a minority of individuals with *cagA*-positive *H. pylori *infection develop severe outcomes in both countries. This suggests that other virulence factors in *cagA*-positive strains are necessary to develop severe outcomes.

Previous whole *H. pylori *genome microarray data revealed that several genes were associated with the presence of *cagA *and/or clinical outcomes. For example, Romo-González *et al*. examined 42 *H. pylori *strains and found that several genes were associated with gastroduodenal diseases [16]. In addition, Salama *et al*. used the microarray of 15 *H. pylori *strains and identified several genes that correlated with the presence of *cag *PAI [17], although they did not examine the association between these genes and clinical outcomes. However, these microarray data are not sufficient as conclusive evidence of the association due to the small sample size. Previously, we also performed whole *H. pylori *genome microarray and examined 1,531 genes, including *cagA*, in 56 *H. pylori *strains isolated from several countries [18].

In this study, we aimed to find novel candidate virulence genes that correlate with the presence of *cagA*, and we examined the association of these genes with clinical outcomes in Colombian and Japanese populations.

## Methods

### Microarray experiments

Initially, candidate genes were selected from previous studies by Salama *et al*. [17] and Romo-González *et al*. [16]. Microarray data from 56 strains in our previous report was then used for the examination of the association of candidate virulence genes with the presence of *cagA *[18].

### Patients

*H. pylori *strains were obtained from the gastric mucosa of *H. pylori*-infected patients who underwent endoscopy at Oita University Faculty of Medicine, Oita, Japan, and Universidad Nacional de Colombia, Bogota, Colombia. Presentations included gastritis, duodenal ulcer (DU), gastric ulcer (GU), and GC. DU, GU, and GC were identified by endoscopy, and GC was further confirmed by histopathology. Gastritis was defined as *H. pylori *gastritis in the absence of peptic ulcers or gastric malignancy. Patients with a history of partial gastric resection were excluded. Patients who received *H. pylori *eradication therapy or treatment with antibiotics, bismuth-containing compounds, H2-receptor blockers, or proton pump inhibitors within 4 weeks prior to the study were also excluded. Informed consent was obtained from all participants, and the protocol was approved by the ethics committees of Oita University and Universidad Nacional de Colombia.

### *H. pylori *genotyping

Antral biopsy specimens were obtained for the isolation of *H. pylori *using standard culture methods, as previously described [19]. Chromosomal DNA was extracted from confluent plate cultures expanded from a single colony using a commercially available kit (QIAGEN Inc., Valencia, CA, USA). Two *H. pylori *strains with full-sequenced genomes, 26695 (ATCC 700392) and J99 (ATCC 700824) deposited in the GenBank, were used as control strains. The *cagA *status was determined by polymerase chain reaction (PCR) using primer pair 5'-ACC CTA GTC GGT AAT GGG-3' and 5'-GCT TTA GCT TCT GAY ACY GC-3' (Y = C+T), as described previously [20]. The *vacA *genotyping (s1, s2, m1, and m2) was performed by PCR, as described previously [21,22]. Primers for the signal region yielded a fragment of 259 bp for s1 variants and one of 286 bp for s2 variants. Primers for the middle region yielded a fragment of 570 bp for m1 variants and one of 645 bp for m2 variants.

Two primer sets for each candidate gene were designed with software Primer 3 (version. 0.4.0) based on the published sequences of *H. pylori *(Table 1). Amplification of *H. pylori *genomic DNA sequences was carried out in a total volume of 25 μL containing 2.5 μL of PCR buffer, 0.2 mM of each deoxynucleotide, 0.625 U of Blend Taq DNA polymerase (Blend Taq, Toyobo Co., Ltd., Osaka, Japan), 0.2 μM of each primer, and more than 10 ng of *H. pylori *DNA. Each reaction mixture was amplified as follows: initial denaturation at 94°C for 5 min, which was followed by 30 cycles of denaturation at 94°C for 30 s, annealing at the indicated temperature in Table 1 for 30 s, extension at 72°C for 1 min, and then final extension at 72°C for 5 min. The amplified fragment was detected by 2.0% agarose gel electrophoresis using an ultraviolet transilluminator.

**Table 1:** Primer sequences

Primer Name	Primer Sequence (5'-3') Forward	Primer Sequence (5'-3') Reverse	AT (°C)	Product size
*hp0967*-1	CATGGCTTTAAATGGCAACA	CCGGCATTAAATCGTTGTTT	58	169
*hp0967*-2	TAGCGTGTATTTTGGCGATG	GATAGCCGGCATTAAATCGT	59	151
*jhp0045*-1	TGGCAAAAGAGTCCAAGACA	CGTTGCAATAAAAACGCAGA	59	180
*jhp0045*-2	AAAACAACGCCTGGTATTGC	GATTGCACTTTATGCGTGTGA	60	158
*jhp0046*-1	AAGCAAGCGATAATGTCATGG	AATTGAGCGTTTTGGTGTCC	59	154
*jhp0046*-2	AGCAAGCGATAATGTCATGG	AATTGAGCGTTTTGGTGTCC	58	153
*jhp0951*-1	CAAAGCGTGAATGATTTGGA	AGATTGCGCAAGGATTTGAG	56	194
*jhp0951*-2	ATGCGTGGCTAAGCGATACT	GACCCAACGCTCTTGAAGTT	57	243

AT; annealing temperature

### Dot blot

For each sample, 500 ng of total DNA was added to 100 μL of TE buffer and mixed with 100 μL of a denaturing buffer (0.5 M NaOH; 1.5 M NaCl). The denatured DNA was transferred to a Hybond-N^+ ^membrane (GE HealthCare, Piscataway, NJ, USA) by means of a Bio-Dot Microfiltration Apparatus (Bio-Rad Laboratories, Inc., Hercules, CA, USA). DNA of J99 and human DNA were also transferred to the membrane and used as positive and negative controls, respectively. The membranes were hybridized at 42°C overnight in plastic bags containing ECL Gold hybridization buffer supplemented with 5% (wt/vol) blocking agent and 0.5 M NaCl. The membranes were washed 3 times in primary washing buffer (0.5× SSC [1× SSC is 0.15 M NaCl plus 0.015 M sodium citrate] [pH 7.0], 0.4% sodium dodecyl sulfate) at room temperature for 15 min and 3 times in secondary washing buffer (2× SSC) at room temperature for 15 min. Finally, the membranes were exposed to Hyperfilm ECL film (GE HealthCare). Gene status was considered positive when at least one of the PCR reactions was positive. When gene status was considered negative by PCR, we further confirmed the results using dot-blot analyses. If PCR results yielded negative results but the dot blot showed a positive blot, we considered the samples positive.

### DNA sequencing

DNA sequencing for the full length of *jhp0045 *(1,032 bp) was performed with several primer pairs located at *jhp0044 *and *jhp0046*. Likewise, DNA sequencing for the full length of *jhp0046 *(783 bp) was performed with several primer pairs located at *jhp0045 *and *jhp0047*. PCR products were purified with Centri-sep Columns (Applied Biosystems by Life Technologies, Tokyo, Japan), and the amplified fragments were sequenced with Hi-Di Formamide (Applied Biosystems by Life Technologies) using an ABI Prism 310 Genetic Analyzer (Applied Biosystems by Life Technologies, Carlsbad, CA, USA) in accordance with the manufacturer's instructions.

### Statistical analysis

Variables such as gender, mean age, and the presence of each candidate gene and *cagA *were evaluated. The univariate association between each genotype and the clinical outcomes were quantified by the chi-square test. A multivariate logistic regression model was used to calculate the odds ratios (OR) of the clinical outcomes by including age, sex, and *H. pylori *genotypes. All determinants with P values of < 0.10 were entered together in the full model of logistic regression, and the model was reduced by excluding variables with P values of > 0.10. ORs and 95% confidence intervals (CIs) were used to estimate the risk. Spearman rank coefficients (*r*) were also determined to evaluate the association between the different genotypes of the strains. A P value of less than 0.05 was accepted as statistically significant. The SPSS statistical software package version 18.0 (IBM Corporation, Armonk, NY, USA) was used for all statistical analyses.

## Results

### Selection of candidate genes

Twelve genes that were strongly associated with *cagA *status in the report by Salama *et al*. [17] were selected. In addition, 26 genes that were associated with severe gastric diseases in the report by Romo-González *et al*. [16] were selected. Because *hp1426 *was reported in both reports, a total of 37 genes were selected as candidate genes, as shown in additional file 1.

Among the 37 genes, the status of 9 genes *(hp0186, hp0713, hp0967, hp1409, hp1410, jhp0045, jhp0046, jhp0950*, and *jhp0951*) were significantly correlated with the *cagA *status in our microarray data [18] (P = 0.026, 0.026, 0.014, 0.048, 0.030, 0.033, 0.033, 0.017, and 0.005 for each above gene, respectively). Among the 9 genes, 4 genes *(hp0967, jhp0045, jhp0046*, and *jhp0951*) were selected in our analyses because the functions of these genes are known. The presence of 3 genes (*hp0967, jhp0045*, and *jhp0046*) was inversely correlated with the presence of *cagA*, but that of *jhp0951 *was positively correlated with the presence of *cagA*. We examined the presence of these candidate genes in 28 full sequenced strains deposited in Genbank. The *hp0967, jhp0045, jhp0046*, and *jhp0951 *were found in 18, 7, 7, and 13 strains, respectively.

### Prevalence of candidate genes in Japan and Colombia

The distribution of the status of *cagA, vacA, hp0967, jhp0045, jhp0046*, and *jhp0951 *in the two countries is shown in Table 2. Three samples from Colombia showed the positive for both *vacA *m1 and m2 genotypes, which suggest the mixed infection, were excluded in the final analysis. Finally, a total of 343 patients were included in this study: 174 from Colombia (68 with gastritis, 43 with DU, and 63 with GC) and 169 from Japan (49 with gastritis, 50 with DU, 50 with GU, and 20 with GC). The results from PCR and dot blot matched well: there were no cases with negative results by PCR and only positive results by dot blot. There were significant differences in the status of *cagA, hp0967, jhp0045*, and *jhp0046 *between strains isolated from Japanese and Colombian populations. The prevalence of *cagA *was 100% in Japan, whereas it was 65.5% in Colombia (P < 0.0001). Higher prevalences of *vacA *s1 and m1 genotypes were found in Japan than Colombia (100 vs. 76.8%, P < 0.001; 100 vs. 62.7%, < 0.001, respectively). The prevalence of *hp0967 *was significantly higher in Japan than Colombia (62.1 vs. 48.0%, P = 0.013). However, the prevalences of *jhp0045 *and *jhp0046 *were more prevalent in Colombia than Japan (23.7 vs. 8.9%, P < 0.0001; 28.2 vs. 8.9%, P < 0.0001, respectively). There was no difference in the prevalence of *jhp0951 *between the 2 countries.

**Table 2:** Relationship between each gene and clinical outcomes in Colombia

	Total		gastritis		DU			GC		
n	174		68		43		P	63		P
mean age	53.4 ± 16.4		52.0 ± 16.5		48.4 ± 15.9		0.50	58.1 ± 15.5		0.01
male	90	(50.8%)	28	(40.6%)	22	(50.0%)	0.30	40	(62.5%)	0.01
*cagA*	116	(65.5%)	44	(63.8%)	27	(61.4%)	0.83	45	(70.3%)	0.41
*vacA *s1	136	(76.8%)	50	(72.5%)	33	(75.0%)	0.70	54	(84.4%)	0.08
*vacA *m1	111	(62.7%)	38	(55.1%)	26	(59.1%)	0.63	48	(75.0%)	0.01
*hp0967*	85	(48.0%)	33	(47.8%)	20	(45.5%)	0.83	32	(50.0%)	0.79
*jhp0045*	42	(23.7%)	14	(20.3%)	13	(29.5%)	0.24	15	(23.4%)	0.65
*jhp0046*	50	(28.2%)	18	(26.1%)	15	(34.1%)	0.34	17	(26.6%)	0.94
*jhp0951*	102	(57.6%)	40	(58.0%)	26	(59.1%)	0.86	36	(56.3%)	0.84

DU, duodenal ulcer; GC, gastric cancer

*P value was compared with gastritis

### The association between candidate genes and clinical outcomes in Colombia

The prevalence of each gene was examined according to clinical outcomes. The prevalence of the *vacA *m1 genotype was significantly higher in strains from patients with GC than those with gastritis (75.0 vs. 55.1%, P = 0.014) (Table 2). Although it is accepted that *cagA *is an important virulence factor, the prevalence of *cagA *was not different between the strains from patients with DU, GC, and gastritis in Colombia (61.4, 70.3, and 63.8%, P > 0.05), which was in agreement with our previous study [23]. Therefore, we hypothesized that the presence/absence of novel factors that accompany the presence of *cagA *leads to severe clinical outcomes in the Colombian population. Based on this hypothesis, the prevalences of these 4 candidate genes were examined in the *cagA*-positive cases. In *cagA*-positive cases, *vacA *status was not associated with clinical outcomes. Interestingly, the prevalence of *jhp0045 *in *cagA*-positive cases from GC was significantly higher than that of gastritis (30.4 vs. 11.4%, P = 0.023) (Table 3). The prevalence of *jhp0046 *in *cagA*-positive cases from GC also tended to be higher than that of gastritis (34.8 vs. 18.2%, P = 0.06), although this did not reach statistical significance. However, there were no associations of these candidates between gastritis and DU. Table 4 shows the association determined by a multivariate analysis between clinical outcomes and the presence of *jhp0045 *or *jhp0046 *in *cagA*-positive cases in the Colombian population. After adjustment for age and gender, *jhp0045 *was an independent factor for discriminating GC from gastritis in *cagA*-positive cases (adjusted OR = 3.24; 95% CI = 1.00-10.42; Table 4). Likewise, *jhp0046 *was an independent factor for discriminating GC from gastritis in *cagA*-positive cases (adjusted OR = 3.16; 95% CI = 1.05-9.47). On the other hand, the prevalences of *hp0967 *and *jhp0951 *in *cagA*-positive cases were not associated with clinical outcomes.

**Table 3:** Relationship between each gene and clinical outcomes in *cagA*-positive cases in Colombia

	Gastritis		DU			GC		
n	44		27		P	45		P
mean age	51.0 ± 15.3		47.2 ± 14.3		0.51	57.6 ± 14.3		0.02
male	19	(43.2%)	13	(46.4%)	0.68	31	(67.4%)	0.02
*vacA *s1	42	(95.5%)	24	(85.7%)	0.27	42	(91.3%)	0.51
*vacA *m1	33	(75.0%)	19	(67.9%)	0.66	36	(78.3%)	0.37
*hp0967*	24	(54.5%)	12	(42.9%)	0.40	27	(58.7%)	0.60
*jhp0045*	5	(11.4%)	7	(25.0%)	0.10	14	(30.4%)	0.02
*jhp0046*	8	(18.2%)	7	(25.0%)	0.43	16	(34.8%)	0.06
*jhp0951*	25	(56.8%)	19	(67.9%)	0.25	27	(58.7%)	0.76

DU, duodenal ulcer; GC, gastric cancer

*P value was compared with gastritis

**Table 4:** Multivariate analyses of the risk for GC by age, gender, and *jhp0045 *or *jhp0046 *status in *cagA*-positive cases in Colombia

	Adjusted OR	95% CI	P
Age (per 1 year)	1.02	0.99-1.05	0.10
Gender (male)	3.19	1.29-7.89	0.01
*jhp0045*	3.24	1.00-10.42	0.04

Age (per 1 year)	1.02	0.99-1.05	0.11
Gender (male)	3.15	1.27-7.80	0.01
*jhp0046*	3.16	1.05-9.47	0.03

CI, confidence interval; GC, gastric cancer; OR, odds ratio

### The association between candidate genes and clinical outcomes in Japan

All samples from Japan showed *cagA*-positive and *vacA *s1m1. The prevalences of the 4 candidate genes in *cagA*-positive cases were independent of clinical outcomes (Table 5).

**Table 5:** Prevalence of each gene and relationship between each gene and clinical outcomes in Japan

	Total		gastritis		DU			GU			GC		
n	169		49		50		P	50		P	20		P
mean age	60.0 ± 12.8		59.3 ± 12.8		56.0 ± 13.6		0.19	62.9 ± 12.1		0.25	64.3 ± 9.4		0.13
male	91	(53.8%)	24	(49.0%)	29	(58.0%)	0.36	27	(54.0%)	0.61	11	(55.0%)	0.65
*cagA*	169	(100.0%)	49	(100.0%)	50	(100.0%)	-	50	(100.0%)	-	20	(100.0%)	-
*vacA *s1	169	(100.0%)	49	(100.0%)	50	(100.0%)	-	50	(100.0%)	-	20	(100.0%)	-
*vacA *m1	169	(100.0%)	49	(100.0%)	50	(100.0%)	-	50	(100.0%)	-	20	(100.0%)	-
*hp0967*	105	(62.1%)	30	(61.2%)	31	(62.0%)	0.93	34	(68.0%)	0.48	10	(50.0%)	0.39
*jhp0045*	15	(8.9%)	2	(4.1%)	6	(12.0%)	0.14	5	(10.0%)	0.22	2	(10.0%)	0.32
*jhp0046*	15	(8.9%)	2	(4.1%)	6	(12.0%)	0.14	5	(10.0%)	0.22	2	(10.0%)	0.32
*jhp0951*	108	(63.9%)	31	(63.3%)	30	(60.0%)	0.73	35	(70.0%)	0.47	12	(60.0%)	0.80

DU, duodenal ulcer; GU, gastric ulcer; GC, gastric cancer

### Sequence analysis of *jhp0045 *and *jhp0046 *in Colombia and Japan

In order to clarify whether the sequence variants in *jhp0045 *and *jhp0046 *contributed to the different outcomes in Colombia and Japan, sequences of these 2 genes were compared using 8 randomly selected strains. For *jhp0045*, one-point mutation in the Japanese strains was found at 643-bp position of J99 (A643G). Therefore, the amino acid was changed from Ile to Val. The sequence of *jhp0045 *from the Colombian strains matched with J99. On the other hand, there was no difference in the sequence of *jhp0046 *between the strains from the 2 countries.

### Nucleotide sequence accession numbers

The nucleotide sequences of *jhp0045 *and *jhp0046 *for 8 strains (Japanese strains: 01-401, 04-156, 05-262, and 07-238; Colombian strains: Colombia 64, Colombia 114, Colombia 174, and Colombia 229) have been deposited in the GenBank database under accession no. AB647162 to AB647169 for *jhp0045 *and AB647170 to AB647176 for *jhp0046*, respectively.

## Discussion

Our study revealed that *jhp0045 *and *jhp0046 *were independent discriminating factors for GC from gastritis in *cagA*-positive cases in Colombia. This suggests that *jhp0045 *and *jhp0046 *play a role in high-risk subjects, such as *cagA*-positive *H. pylori*-infected cases.

Several genes of *H. pylori *were reported as virulence factors, and these include *cagA, vacA, oipA, babA, hopQ*, and *homA/B *[4-10]. Importantly, most virulence factors correlated with each other; *cagA*-positive strains also possess the *vacA *s1/m1 genotype, and this is closely linked to the presence of the *babA *and *oipA *"on" status [14]. Therefore, we hypothesized that undefined novel virulence genes could exist in genes correlated with *cagA *status. Although previous microarray data showed that several genes correlated with *cagA *status, the sample number in these microarray reports was not enough to be conclusive (eg, [15] strains in the report by Salama *et al*. [17]). Among the 37 genes we selected, 9 genes were significantly correlated with the presence of *cagA*. In the present study, we focused on 4 genes whose functions have been revealed. *hp0967 *is considered a virulence-associated protein D, and *jhp0951*, which encodes an integrase of the XerCD family, has been reported to be related to modifications in the response to low pH and iron limitations [16,24]. The putative functions of *jhp0045 *and *jhp0046 *have been described as type-II DNA methyltransferase and type-II restriction enzymes, respectively [17,25].

Among these 4 genes, *jhp0045 *and *jhp0046 *were significantly associated with severe clinical outcomes in *cagA*-positive cases in Colombia. Although *hp0967 *has been reported to be negatively associated with DU [16], there was no association in this study. The *jhp0951 *has been positively associated with DU [16]; however, an association was not found in this study. These findings suggest that these microarray data are not conclusive. A larger group of subjects is necessary to clarify the association.

The mechanisms of the development of GC in those patients infected with *jhp0045 *or *jhp0046 *are unclear, although the putative functions of *jhp0045 *and *jhp0046 *have been described as a type-II DNA methyltransferase and a type-II restriction enzyme, respectively [17,25]. In this study, most strains possessing *jhp0045 *had *jhp0046*. This suggests that these 2 genes may work together. This combination of a restriction enzyme and a methyltransferase is known as a restriction-modification (R-M) system [26]. It has been reported that *H. pylori *possess an extraordinary number of genes with homology to R-M genes in other bacterial species [25,27,28]. However, not all R-M systems have that function. Kong *et al*. reported that, among the 16 completely tested Type II R-M systems in J99, only 4 were fully functional in that they contained both active endonucleases and methylases [29]. The *jhp0045 *and *jhp0046 *were included in these functional ones. Because several R-M systems are correlated with pathogenicity [26], strains possessing *jhp0045 *and *jhp0046 *may be considered as truly virulent strains. Interestingly, recent reports showed that *H. pylori *strains possessing *cagA *from Colombia can be divide into 2 groups by 7 housekeeping genes [30]. This grouping was related with severe histological scores and the prevalence of GC. The *jhp0045 *and *jhp0046 *may be a discriminating factor for this grouping. Further study is necessary in order to examine the relationship between *jhp0045 *and *jhp0046 *and the grouping by 7 housekeeping genes.

Only 1 change of an amino acid resulted from a point mutation of *jhp0045 *in Japanese strains compared with Colombian strains and J99. It is not clear whether this difference contributed to the different results between the 2 countries. Variants of virulence factors in different areas may result in different clinical outcomes. For example, *cagA *can be divided into 2 types (East-Asian-type *cagA *and Western-type *cagA*) according to differences in the 3' region [6,20,21]. Some reports have shown that individuals infected with East-Asian-type *cagA *strains have an increased risk of peptic ulcer or GC compared to those infected with Western-type *cagA *strains [31,32]. Further studies to clarify the mechanisms or functions according to the different amino acid sequences are necessary to explain this.

Our study had several limitations. First, not only the *H. pylori *virulence factors, but also environmental factors (eg, diet) and host factors have been demonstrated to be predictors of severe clinical outcomes [33]. Especially, inflammatory cytokine gene polymorphisms (*IL-1 *gene cluster, *TNF-α, IL-10*, and *IL-8*) have been reported to be correlated with gastric cancer [34-39]. Further study will be necessary in order to elucidate the role of our candidate genes of *H. pylori*. Second, we did not examine known virulence factors other than *cagA *and *vacA *of *H. pylori*. It is possible that our candidate genes might correlate with other known virulence factors, even in *cagA*-positive cases. It is better to examine host factors and other known virulence factors in order to clarify the role of our candidate genes in future studies. Finally, we examined the status of the genes by only positivity or negativity. The levels of gene expression can be affected by clinical outcomes. In addition, gene expression is not always correlated with protein expression patterns. For example, the expression of the blood group antigen-binding adhesin (BabA) protein is not always correlated with *babA *gene expression [40]. Further analysis using real-time PCR or immunoblotting techniques is necessary to clarify the significance of our candidate genes.

## Conclusions

The *jhp0045 *and *jhp0046 *were associated with GC in *cagA*-positive cases in Colombia but not in Japan. In Colombia, the status of *jhp0045 *and *jhp0046 *may predict the future development of GC for patients with gastritis. A prospective study is necessary to confirm this. Moreover, the study of the distribution of these genes in other populations would be interesting in order to further elucidate the associations found in the present study and the possible virulence role of these factors in *H. pylori *infection.

## Competing interests

The authors declare that they have no competing interests.

## Authors' contributions

Conceived and designed the experiments: MW SS YY. Performed experiments: MW OM. Analyzed the data: MW SS RS YY. Contributed reagents/materials/analysis tools: MW OM SS YY KM TF. Wrote the paper: MW SS YY. All authors read and approved the final manuscript.

## Pre-publication history

The pre-publication history for this paper can be accessed here:

http://www.biomedcentral.com/1471-230X/11/141/prepub

## Supplementary Material

Additional file 1 **Selected 37 genes**. A total of 37 genes were selected as candidate genes from two reports (Salama *et al*. [17] and Romo-González *et al*. [16]). Click here for file
